# Vitamin D supplementation may be beneficial in improving the prognosis of patients with chronic obstructive pulmonary disease in the intensive care unit: a retrospective study

**DOI:** 10.3389/fmed.2024.1334524

**Published:** 2024-03-22

**Authors:** Qian He, Song Hu, Jun Xie, Yunqi Ge, Chong Li

**Affiliations:** Department of Respiratory and Critical Care Medicine, Third Affiliated Hospital of Soochow University, Changzhou, China

**Keywords:** vitamin D, chronic obstructive pulmonary disease, intensive care unit, mortality, exacerbations, respiratory failure

## Abstract

**Background:**

Vitamin D is a crucial fat-soluble vitamin that has garnered significant attention due to its potential impact on respiratory health. It is noteworthy that many patients with chronic obstructive pulmonary disease (COPD) often experience deficiencies or insufficiencies of vitamin D. To address this issue, our retrospective study aimed to explore the potential association between serum 25-hydroxyvitamin D concentration and the prognoses of COPD patients in the Intensive Care Unit (ICU).

**Methods:**

This study utilised data from the Medical Information Marketplace in Intensive Care IV (MIMIC-IV), a database of patients admitted to the Intensive Care Unit at Beth Israel Deaconess Medical Center (BIDMC) in the United States of America, with a focus on patients with a diagnosis of COPD. These patients were categorized into two groups: those who received vitamin D supplementation during their ICU stay and those who did not. We assessed in-hospital mortality and ICU mortality outcomes. Our analysis involved various analytical tools, including Kaplan–Meier survival curves, Cox proportional risk regression models, and subgroup analyses, to investigate the relationship between vitamin D supplementation and these outcomes. Additionally, we employed propensity-score matching (PSM) to enhance the reliability of our findings.

**Results:**

The study included a total of 3,203 COPD patients, with 587 in the vitamin D group and 2,616 in the no-vitamin D group. The Kaplan–Meier survival curve demonstrated a significant difference in survival probability between the two groups. After adjusting for potential confounders using Cox regression models, the vitamin D group exhibited a substantially lower risk of in-hospital and ICU mortalities compared to the no-vitamin D group. The hazard ratios for in-hospital and ICU mortalities in the vitamin D group were 1.7 (95% CI: 1.3, 2.3) and 1.8 (95% CI: 1.2, 2.6), respectively. Propensity-score matching (PSM) estimation yielded consistent results. Furthermore, in the subgroup analysis, female patients who received vitamin D supplementation showed a reduced risk of in-hospital mortality.

**Conclusion:**

The study suggests that vitamin D supplementation may be linked to a reduction in in-hospital and ICU mortalities among COPD patients in the ICU. Of particular note is the potential benefit observed in terms of in-hospital mortality, especially for female patients.

## Background

COPD is a progressive respiratory disorder characterized by airflow limitation and increased susceptibility to exacerbations (1). Patients with severe COPD often require intensive care unit (ICU) admission due to acute exacerbations or respiratory failure. Despite advancements in critical care management, the mortality rate among ICU-admitted COPD patients remains high (2). Identifying modifiable factors that influence the outcome in these patients is crucial for improving their prognosis.

Vitamin D is a fat-soluble vitamin well-known for its crucial roles in calcium metabolism, bone health, and immune function. Moreover, emerging evidence suggests that vitamin D may play an essential role in respiratory health and disease outcomes (3–5). Vitamin D deficiency is highly prevalent among COPD patients, primarily due to limited sun exposure, impaired synthesis, and altered metabolism (6). Several studies have shown a strong association between vitamin D and the risk of developing COPD, disease severity and acute exacerbations (7, 8).

The potential mechanisms underlying the influence of vitamin D on mortality in COPD patients are multifactorial. Vitamin D regulates the immune response, modulates inflammation, and enhances antimicrobial defenses, which may help reduce the risk of respiratory infections and subsequent mortality (9). Moreover, vitamin D exerts direct effects on lung tissue and respiratory muscle function, possibly influencing disease progression and outcomes (10). However, the relationship between vitamin D supplementation and mortality rates specifically in ICU-admitted COPD patients remains unclear. Therefore, the purpose of this article is to explore the relationship between vitamin D supplementation and mortality in ICU-admitted COPD patients.

## Methods

### Data source

We conducted a study involving patients with COPD using data from the MIMIC-IV (Medical Information Marketplace for Critical Care IV, version 2.0) database of patients admitted to the Intensive Care Unit of the Beth Israel Deaconess Medical Centre (BIDMC), which contains comprehensive data on 315,460 inpatients from 2008 to 2019 (11). Informed consent was waived due to the utilization of publicly available data. However, our research adhered to ethical guidelines, and the primary researcher involved completed the Protecting Human Research Participants online course provided by the National Institutes of Health (certification number: 49872601). Access to the database was approved by the MIT Institutional Review Committee and Beth Israel Deaconess Medical Center. To protect patient privacy, all personally identifiable information within the database has been removed. This study was conducted in accordance with the principles outlined in the Declaration of Helsinki.

### Study population

In the MIMIC-IV database, our study focused on patients who were diagnosed with COPD upon hospital admission. The data extraction process involved identifying cases using specific diagnosis codes from the International Classification of Diseases version 10 (“J44,” “J440,” “J441,” “J449”). The exclusion criteria were as follows: (1) aged <18 years; (2) patients with repeated hospital or ICU admissions; (3) hospital or ICU stay less than 24 h. Based on whether patients received vitamin D supplementation (including both intravenous and oral routes), they were categorized into either the vitamin D group or the no-vitamin D group.

### Data extraction and outcomes

From the MIMIC-IV version 2.0 database, we extracted various variables for analysis. These included demographic characteristics such as age, gender, and weight. We also collected vital signs within 24 h of ICU admission, which encompassed heart rate, mean arterial pressure (MAP), respiratory rate, temperature, and pulse oxygen saturation (SpO2). Laboratory parameters obtained within the same timeframe consisted of hemoglobin, hematocrit, platelet count, anion gap, bicarbonate, chloride, blood urea nitrogen (BUN), calcium, glucose, white blood cell (WBC) count, red blood cell (RBC) count, creatinine, prothrombin time (PT), International Normalized Ratio (INR), serum sodium, and serum potassium. Additionally, we recorded comorbidities such as congestive heart failure (CHF), myocardial infarction, rheumatic disease, peptic ulcer disease, renal failure, diabetes, malignant cancer, and liver disease. To assess the severity of illness, we collected data on Sequential Organ Failure Assessment (SOFA) score, Oxford Acute Severity of Illness Score (OASIS), and Acute Physiology Score III (APS III). Information regarding ventilator use, vasopressor use, and renal replacement therapy (RRT) use was also included. The outcomes of this study were in-hospital and ICU mortality.

### Statistical analysis

We excluded variables with more than 10% missing values from the analysis. For the remaining missing data, we used mean imputations to estimate their values. As the continuous variables in our study did not follow a normal distribution, we expressed them as median and interquartile range (IQR) values. We employed the Mann–Whitney U test to assess the differences between the two groups. Categorical variables were presented as frequencies or percentages, and their analysis was conducted using the Chi-square or Fisher’s exact test.

To evaluate the impact of vitamin D administration on patient survival, we used Kaplan–Meier (KM) curves and log-rank tests. Additionally, we employed multivariate Cox regression models to estimate the association between vitamin D supplementation and mortality in COPD patients. Model 1 did not adjust for any covariates, while Model II was adjusted for age and gender. Model III included adjustments for age, gender, hematocrit, hemoglobin, platelets, WBC, RBC, anion gap, bicarbonate, BUN, calcium, chloride, creatinine, sodium, potassium, glucose, weight, heart rate, MAP, respiratory rate, temperature, SpO2, INR, PT, RRT, vasopressor use, ventilator use, APS III, OASIS, SOFA, congestive heart failure, myocardial infarction, rheumatic disease, peptic ulcer disease, renal disease, diabetes, malignant cancer, and liver disease.

To ensure the robustness of the findings, propensity-score matching (PSM) was conducted to minimize baseline differences between the two groups. PSM was performed at a 1:1 ratio using a caliper width of 0.02 standard deviations of the logit of the propensity score. Statistical analysis was carried out using R software (version 4.2.3) and IBM SPSS Statistics (version 23.0; Armonk, NY, United States). A significance level of *p* < 0.05 was considered statistically significant.

## Results

### Baseline characteristics

**Figure 1:**
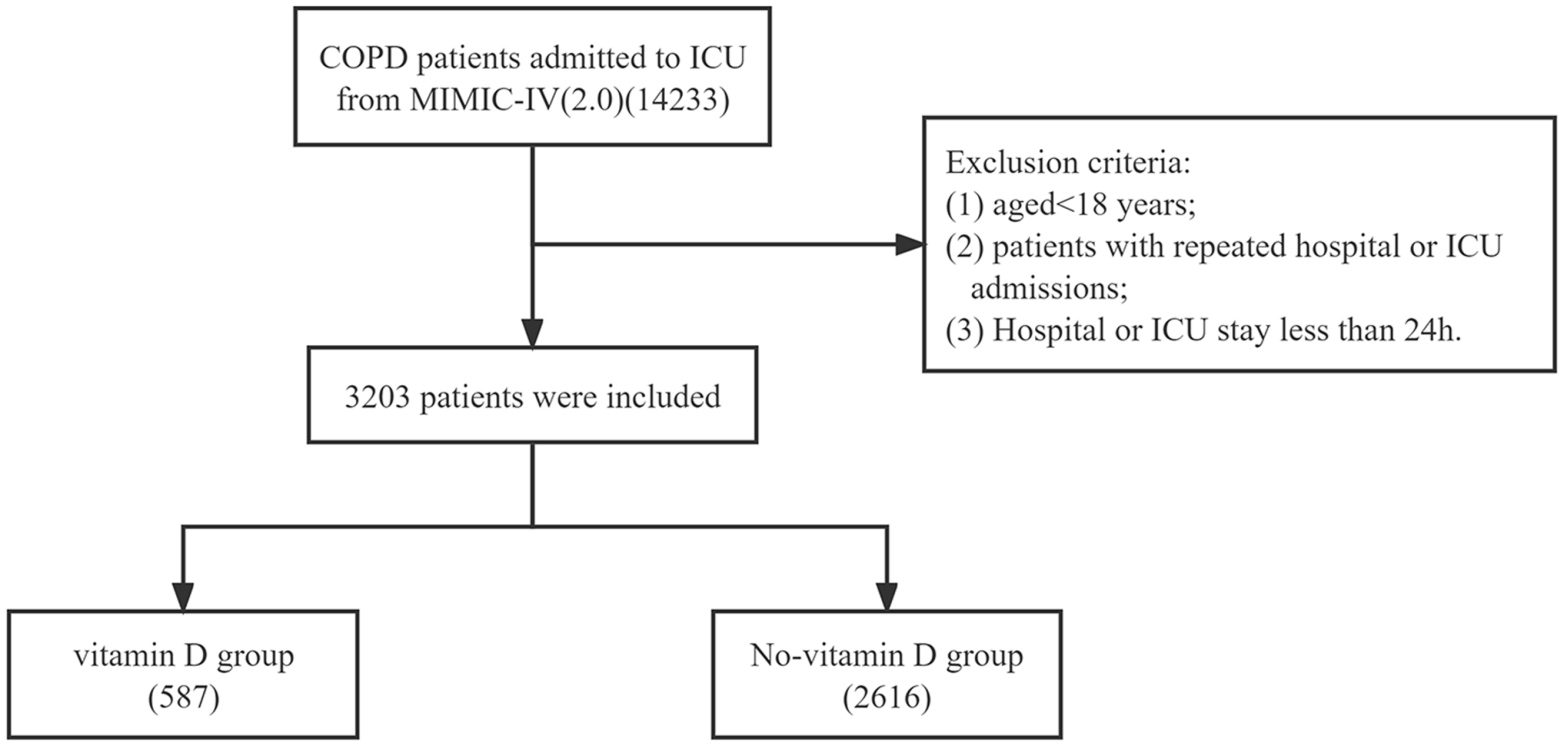
The flow chart of the included population.

A total of 3,203 patients with COPD were included in this study (Figure 1). They were divided into two groups based on their use of vitamin D. The vitamin D group consisted of 587 cases, while the no-vitamin D group had 2,616 cases. Table 1 presents the demographic characteristics, vital signs, laboratory indicators, and baseline comorbidity details. In comparison to the no-vitamin D group, the vitamin D group exhibited lower levels of SpO2, hemoglobin, hematocrit, chloride, and OASIS scores. However, the vitamin D group had higher weight, bicarbonate, BUN, calcium, creatinine, PT, and APS III score. The use of ventilators was more frequent in the non-vitamin D group. Regarding patient outcomes, the no-vitamin D group had significantly higher in-hospital and ICU mortality rates.

**Table 1:** Baseline characteristics of the original population.

	Vitamin D	No vitamin D	*p*
*N*	587	2,616	
Age (years)	72 (63,79)	72 (64,80)	0.5
Gender, *n*			
Female	297	1,223	0.09
Male	290	1,393	
Weight (kg)	82 (66,100)	78 (64,95)	0.009
Vital signs			
HR	84 (74,97)	83 (74,96)	0.4
MAP, mmHg	76 (70,83)	77 (71,84)	0.3
Respiratory rate (breaths/min)	19 (17,22)	19 (17,22)	0.84
Temperature (°C)	37 (36,37)	37 (36,37)	0.9
SpO2, %	96 (94,97)	96 (95,98)	<0.001
Laboratory parameters			
Hemoglobin, g/dL	10 (9,11)	10 (9,12)	<0.001
RBC count, 10^9^/L	3.7 (3.2,4.1)	3.7 (3.2,4.2)	0.06
Hematocrit, %	32 (27,36)	32 (28,37)	0.003
Platelet, 10^9^/L	200 (146,257)	191 (141,252)	0.1
WBC count, 10^9^/L	11 (8,15)	12 (9,15)	0.2
Anion gap, mg/dL	15 (13,17)	15 (12,17)	0.5
Bicarbonate, mg/dL	24 (21,28)	24 (21.27)	0.01
Bun, mg/dL	24 (16,42)	22 (15,36)	0.001
Calcium, mg/dL	9 (8,9)	8 (8,9)	0.005
Chloride, mmol/L	101 (96,105)	102 (98,105)	<0.001
Creatinine mg/dL	1.1 (0.8,1.9)	1.1 (0.8,1.6)	0.01
Glucose, mmol/L	137 (109,170)	132 (110,163)	0.2
Sodium, mg/dL	139 (135,141)	139 (136,141)	0.4
Potassium, mg/dL	4.3 (4.0,4.8)	4.4 (4,4.8)	0.4
INR	1.3 (1.1,1.5)	1.3 (1.1,1.5)	0.02
PT	14 (12,16)	14 (12,16)	0.03
Severity of illness			
SOFA score	5 (2,8)	5 (3,8)	0.8
APS III score	48 (37,63)	46 (34,62)	0.008
OASIS	32 (27,39)	33 (27,40)	0.06
Comorbidities			
Myocardial infarct, *n*	132	690	0.05
Congestive heart failure, *n*	340	1,269	<0.001
Rheumatic disease, *n*	37	118	0.07
Peptic ulcer disease, *n*	17	77	0.4
Liver disease, *n*	60	236	0.9
Diabetes, *n*	250	959	0.007
Renal disease, *n*	212	760	0.001
Malignant cancer, *n*	86	386	0.9
Ventilator use, *n*	167	995	<0.001
RRT use, *n*	46	117	0.001
Vasopressor use, *n*	45	255	0.5
Mortality			
In-hospital mortality, *n*	63	372	0.03
ICU-mortality, *n*	33	259	0.001

INR, international normalized ratio; PT, prothrombin time; RRT, renal replacement therapy.

### Survival analysis and cox proportional-hazards regression model

**Figure 2:**
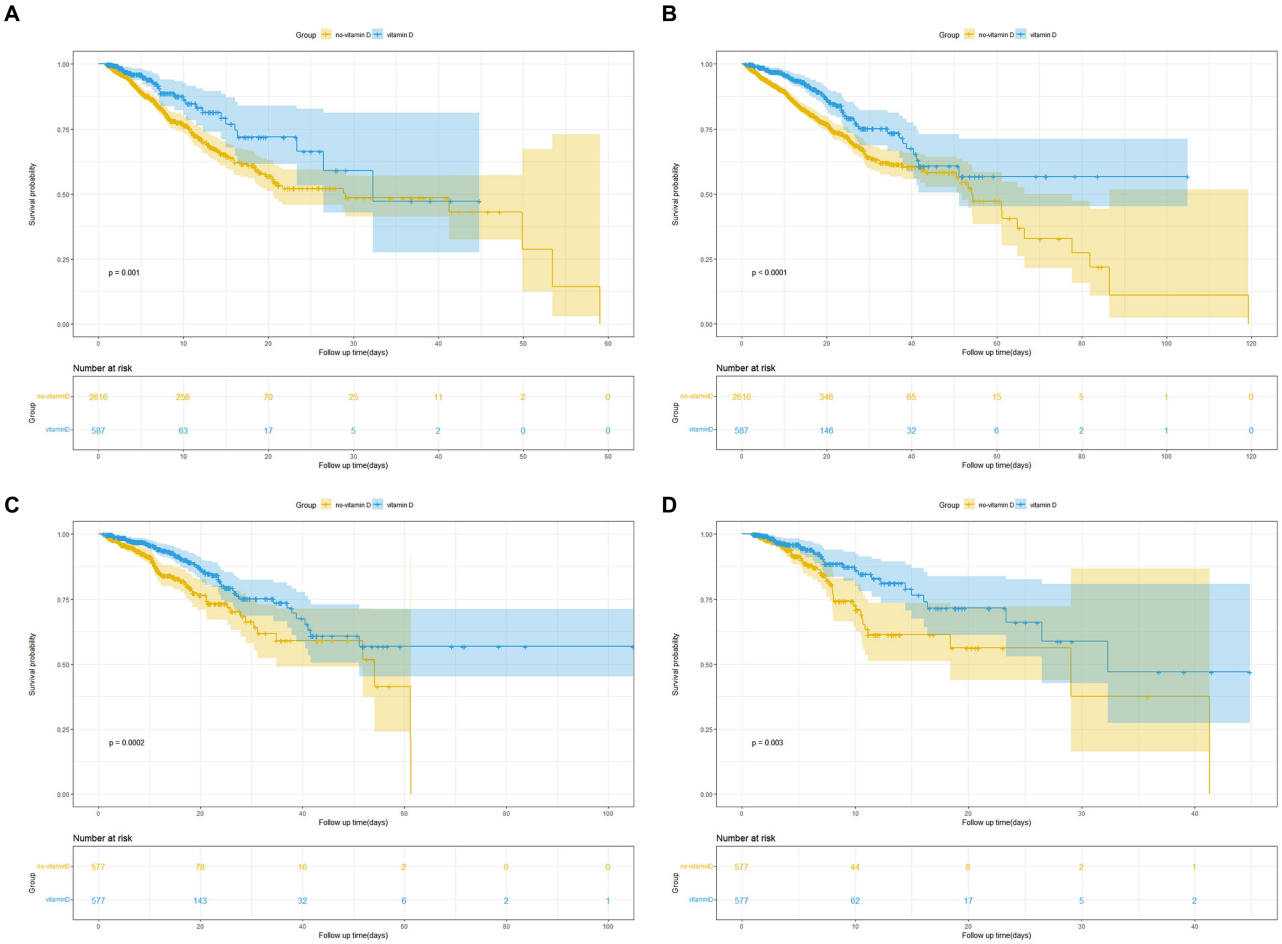
**(A)** Kaplan Meier curve of in-hospital mortality risk in two groups for the original population. **(B)** Kaplan Meier curve of ICU mortality risk in two groups for the original population. **(C)** Kaplan Meier curve of in-hospital mortality risk in two groups for the PSM population. **(D)** Kaplan Meier curve of ICU mortality risk in two groups for the PSM population. (Yellow and blue shaded parts in the figure indicate the 95% confidence interval).

**Figure 3:**
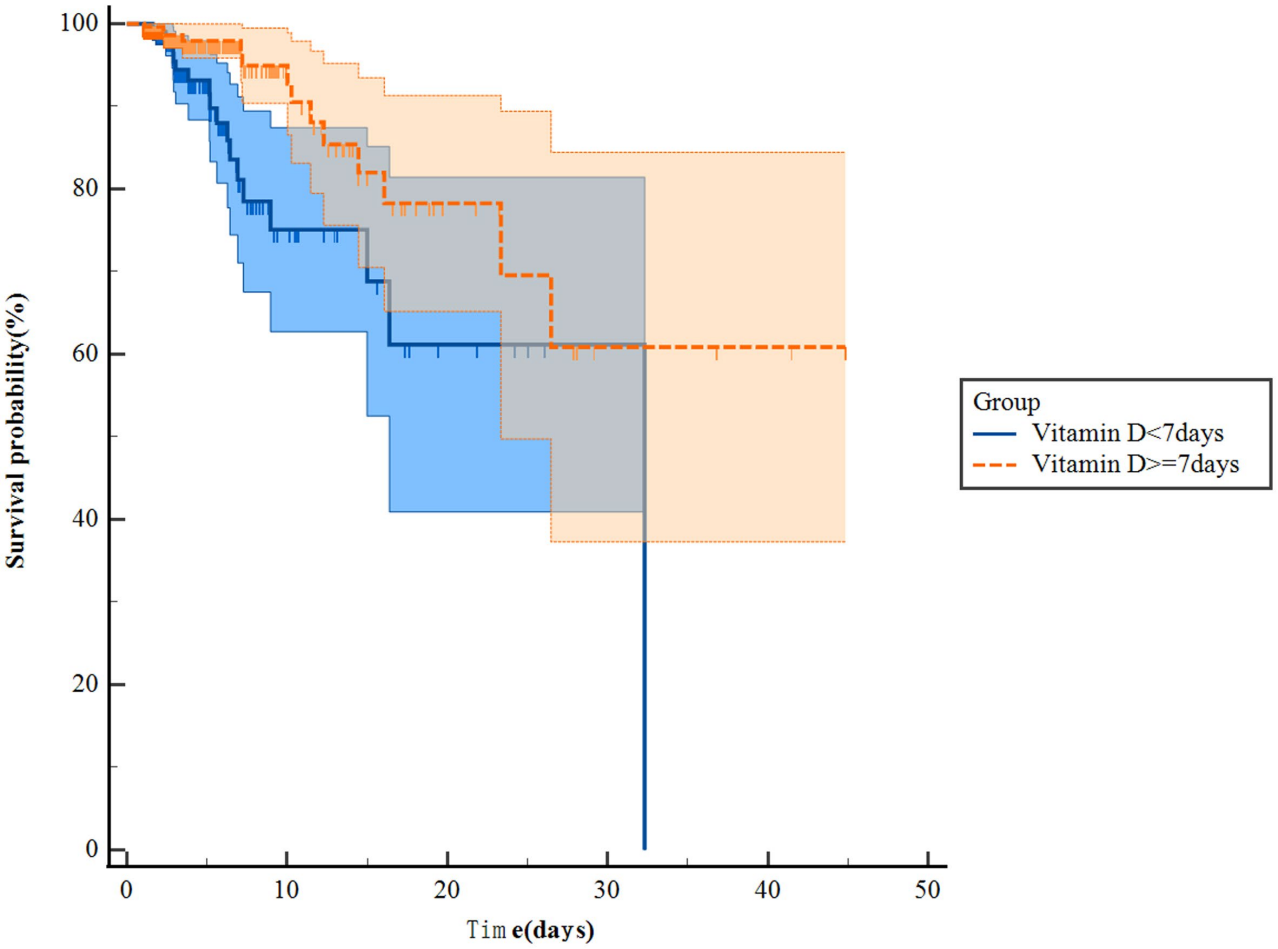
Kaplan Meier curve of ICU mortality risk in two groups for the original population.

The Kaplan–Meier survival curves revealed that patients in the vitamin D group had notably higher in-hospital survival rates compared to those in the no-vitamin D group (*p* < 0.001). This trend was similarly observed in the ICU survival curves (Figure 2). Besides, we further analysis found that the survival probability in the group using vitamin D >7 days increased significantly compared to the group using vitamin D ≤ 7 days (*p* = 0.005, Figure 3). When examining the raw model (Model 1) without any adjustments, it was found that in-hospital (HR 1.9; 95% CI 1.4–2.4; *p* < 0.001) and ICU mortality (HR1.8; 95% CI 1.3–2.6; *p* = 0.001) were significantly higher in the no-vitamin D group compared to the vitamin D group. Model 2, which adjusted for sex and gender, also demonstrated an association between no-vitamin D group and increased in-hospital (HR 1.8; 95% CI 1.4–2.4; *p* < 0.001) and ICU mortality (HR 1.7; 95% CI 1.2–2.5; *p* = 0.003). In Model 3, after adjusting for a range of confounding variables, the mixed-effects Cox proportional hazards models indicated that the no-vitamin D group was associated with a elevated risk of in-hospital (HR 1.7; 95% CI 1.3–2.3; *p* < 0.001) and ICU mortality (HR 1.8; 95% CI 1.6–2.6; *p* = 0.003) when compared to the vitamin D group (Table 2).

**Table 2:** Results of cox proportional hazard models.

Outcomes	Model 1		Model 2		Model 3	
Original population	HR (95% CIs)	*p*-value	HR (95% CIs)	*p*-value	HR (95% CIs)	*p*-value
In-hospital mortality						
No-vitamin D	1.9 (1.4,2.4)	<0.001	1.8 (1.4,2.4)	<0.001	1.7 (1.3,2.3)	<0.001
Vitamin D	1		1		1	
ICU-mortality						
No-vitamin D	1.8 (1.3,2.6)	0.001	1.7 (1.2,2.5)	0.003	1.8.(1.2,2.6)	0.003
Vitamin D	1		1		1	
PSM						
In-hospital mortality						
No-vitamin D	1.8 (1.3,2.6)	<0.001	1.8 (1.3,2.5)	<0.001	2.2 (1.5,3.1)	<0.001
Vitamin D	1		1		1	
ICU-mortality						
No-vitamin D	1.9 (1.2,3.0)	0.004	1.9 (1.2,2.9)	0.006	3.1 (1.8,5.3)	<0.001
Vitamin D	1		1		1	

Model1 covariates were adjusted for nothing. Model2 covariates were adjusted for age and gender. Model3 covariates were adjusted for age, gender, hematocrit, hemoglobin, platelets, WBC, RBC, anion gap, bicarbonate, BUN, calcium, chloride, creatinine, sodium, potassium, glucose, weight, heart rate, MAP, respiratory rate, temperature, SpO2, INR, PT, RRT, vasopressor use, ventilator use, APS III, OASIS, SOFA, congestive heart failure, myocardial infarction, rheumatic disease, peptic ulcer disease, renal disease, diabetes, malignant cancer, and liver disease.

### Propensity score matching

To minimize confounding bias, we conducted propensity score matching (PSM) based on the use of vitamin D. A total of 1,154 pairs of patients were successfully matched, resulting in balanced baseline characteristics between the two groups (Table 3). The Kaplan–Meier survival curves for the matched populations displayed a trend consistent with that observed in the original population (Figures 2C,D). Similar to the original population, we performed multivariate Cox regression analyses on the matched populations. After adjusting for confounding factors, In-hospital mortality (HR 2.2; 95% CI 1.5–3.1; *p* < 0.001) and ICU mortality (HR 3.1; 95% CI 1.8–5.3; *p* < 0.001) were significantly higher in the no-vitamin D group compared with the vitamin D group (Table 2).

**Table 3:** Characteristics of the study population after propensity score matching.

	Vitamin D	No vitamin D	*p*
*N*	577	577	
Age (years)	72 (64,79)	72 (64,79)	0.9
Gender, *n*			
Female	292	306	0.4
Male	285	271	
Weight (kg)	82 (66,99)	80 (66,97)	0.6
Vital signs			
HR	84 (74,97)	83 (74,97)	0.8
MAP, mmHg	76 (70,83)	75 (70,84)	0.4
Respiratory rate (breaths/min)	19 (17,22)	19 (17,22)	0.9
Temperature (°C)	37 (36,37)	37 (36,37)	0.8
SpO2, %	96 (94,97)	96 (94,98)	0.3
Laboratory parameters			
Hemoglobin, g/dL	9.8 (8.5,11.4)	9.7 (8.3,11.3)	0.3
RBC count, 10^9^/L	3.7 (3.2,4.1)	3.6 (3.1,4.1)	0.6
Hematocrit, %	31.7 (27.2,35.9)	30.7 (26.3,35.5)	0.1
Platelet, 10^9^/L	200 (146,256)	201 (141,273)	0.9
WBC count, 10^9^/L	11 (8,15)	12 (8,15)	0.5
Anion gap, mg/dL	15 (13,17)	15 (13,17)	0.9
Bicarbonate, mg/dL	24 (21,28)	24 (22,28)	0.9
Bun, mg/dL	24 (16,41)	24 (16,40)	0.7
Calcium, mg/dL	8.6 (8.1,9)	8.6 (8.2,9.0)	0.4
Chloride, mmol/L	100 (95,105)	101 (96,105)	0.7
Creatinine mg/dL	1.1 (0.8,1.9)	1.1 (0.8,1.8)	0.9
Glucose, mmol/L	136 (109,170)	134 (111,163)	0.8
Sodium, mg/dL	139 (135,141)	138 (136,141)	0.4
Potassium, mg/dL	4.3 (4.0,4.8)	4.3 (4.0,4.7)	0.7
INR	1.3 (1.1,1.5)	1.3 (1.1,1.5)	0.9
PT	14 (12,16)	14 (12,16)	0.9
Severity of illness			
SOFA score	5 (2,8)	5 (2,8)	0.5
APS III score	48 (37,62)	47 (35,62)	0.4
OASIS	32 (27,39)	32 (26,37)	0.4
Comorbidities			
Myocardial infarct, *n*	130	121	0.5
Congestive heart failure, *n*	332	325	0.7
Rheumatic disease, *n*	36	37	0.9
Peptic ulcer disease, *n*	17	25	0.2
Liver disease, *n*	59	74	0.2
Diabetes, *n*	242	231	0.5
Renal disease, *n*	204	191	0.4
Malignant cancer, *n*	86	86	1
Ventilator use, *n*	165	165	1
RRT use, *n*	40	33	0.4
Vasopressor use, *n*	45	38	0.4
Mortality			
In-hospital mortality, *n*	62	81	0.09
ICU-mortality, *n*	33	51	0.04

INR, international normalized ratio; PT, prothrombin time; RRT, renal replacement therapy.

### Subgroup analyses and propensity score matching

**Figure 4:**
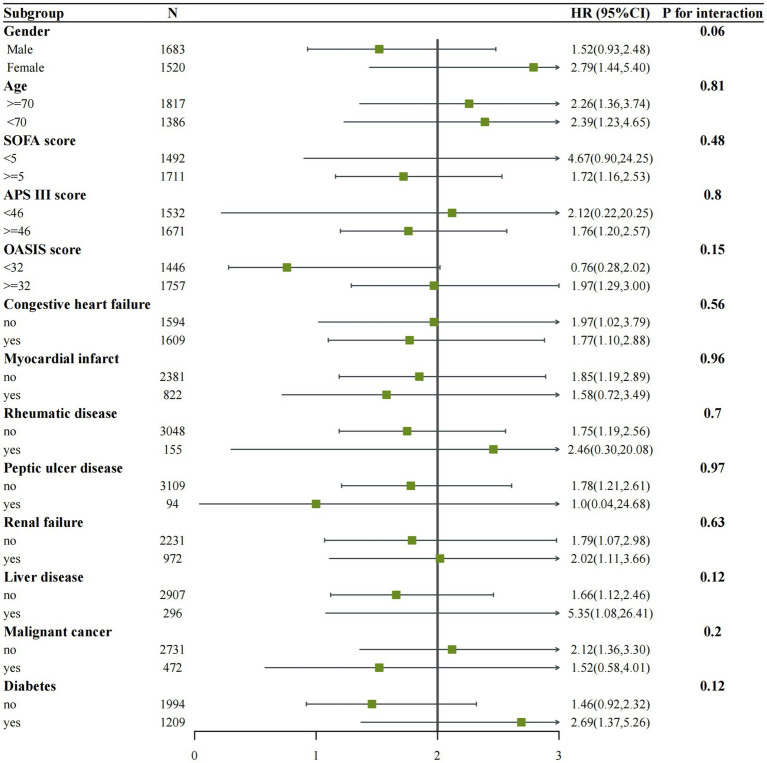
Subgroup analysis of the associations between in-hospital mortality and vitamin D received. Confounders were consistent with the model III in Table 2.

**Figure 5:**
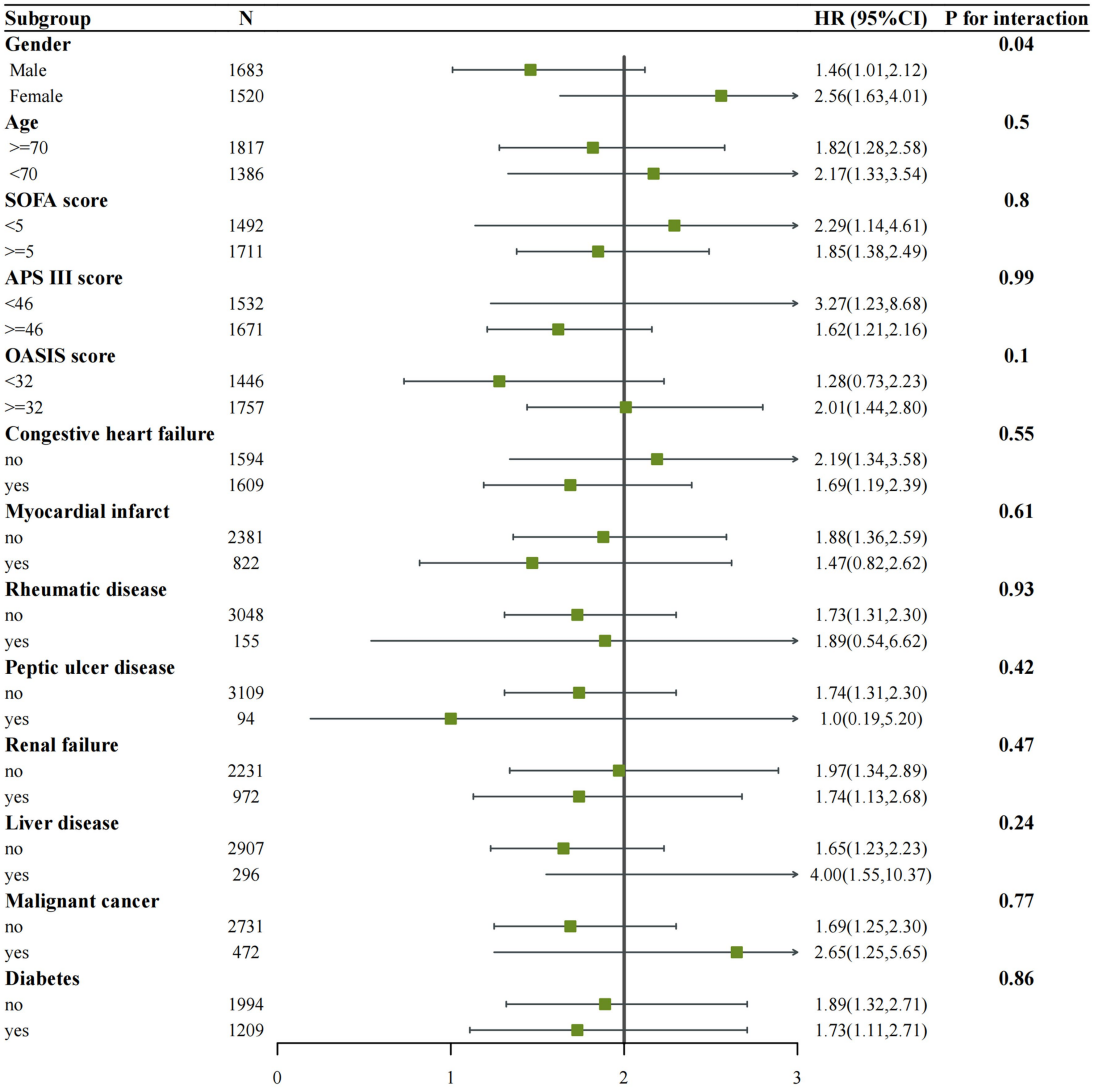
Subgroup analysis of the associations between ICU mortality and vitamin D received. Confounders were consistent with the model III in Table 2.

We performed subgroup analyses of in-hospital and ICU mortality outcomes based on clinically meaningful scores and several comorbidities. Subgroup analyses were performed based on age, gender, congestive heart failure, myocardial infarction, rheumatic disease, peptic ulcer disease, renal failure, liver disease, malignant cancer, diabetes, SOFA score, APS III score, and OASIS score for the outcomes (Figures 4, 5). We observed a significant interaction between gender and in-hospital mortality. The use of vitamin D demonstrated a significant protective effect on female patients. No significant interactions were observed in other stratified populations (*p* for interaction > 0.05).

## Discussion

Based on analysis of the MIMIC-IV database, this study provides the first determination of the effects of vitamin D supplementation on ICU and hospital mortality risk in patients with COPD. The results are highly promising, confirming the association of vitamin D as a low-cost, readily available, and relatively safe drug with improved outcomes for COPD patients. PSM further validates this finding. The stable and reliable results presented herein provide new evidence for clinical treatment research in COPD. The findings of the subgroup analysis suggest that vitamin D use was possible beneficial role in female patients.

Vitamin D, including ergocalciferol (D2) and cholecalciferol (D3), is crucial for bone health and mineral metabolism. It plays a role in regulating the renin-angiotensin-aldosterone system (RAAS) and modulating myocardial extracellular matrix turnover (12). Deficiency in vitamin D can lead to heart function deterioration and accelerate myocardial remodeling (13). One study concluded that serum 25(OH)D concentration are strongly associated with cardiovascular disease and all-cause mortality (14). Moreover, vitamin D insufficiency is associated with conditions such as rickets, osteoporosis, osteomalacia, and other bone-related disorders (15). Recent studies have revealed broader physiological functions of vitamin D, which may have clinical implications for COPD patients (16, 17).

Some experimental studies have highlighted the importance of vitamin D in the growth and development of COPD (18, 19). Vitamin D is found in alveolar type II cells, where it enhances surfactant synthesis and regulates interactions between epithelial and mesenchymal cells (20). Notably, vitamin D deficiency appears to be more prevalent and severe in COPD patients. Research has shown that COPD patients have significantly lower levels of vitamin D, with up to 27% having levels below 4–5 ng/mL (21). A recent meta-analysis examining the relationship between vitamin D and COPD confirmed lower serum 25(OH)D concentration in COPD patients compared to control subjects (22). From a molecular biology perspective, cigarette smoke extracts inhibit the translocation of vitamin D receptors (VDR) in human alveolar epithelial cells, leading to a decrease in local vitamin D signaling (23). Furthermore, COPD patients may have reduced exposure to sunlight, which is necessary for vitamin D synthesis. This is especially true for patients admitted to the ICU, as they often have limited outdoor activities due to respiratory distress. In a study involving hospitalized COPD patients with acute exacerbations, the median 25(OH)D concentration was found to be 7.1 ng/dL, well below normal levels (24). Disease severity has also been shown to correlate negatively with 25(OH)D concentration (25). Vitamin D deficiency may increase bacterial load in the airways, thereby increasing the risk of COPD exacerbations. It is worth noting that most COPD patients experience exacerbations triggered by infections. The production of an antimicrobial peptide called cathelicidin (LL-37) in the airways has been found to be effective against antibiotic-resistant strains like *Staphylococcus aureus*, Chlamydia, and viruses. The genes responsible for producing LL-37 are regulated by regions in their promoters that contain vitamin D receptors. In cases of vitamin D deficiency, the level of LL-37 decreases (26). Consequently, individuals with vitamin D deficiency may have an increased risk of COPD exacerbations due to a higher bacterial load in the airways.

Vitamin D plays a role in stimulating cells of the adaptive immune system, promoting their differentiation towards a tolerogenic phenotype and reducing the production of pro-inflammatory mediators (27). It also enhances phagocytosis and the antimicrobial activity of innate immune cells (28). Vitamin D has been shown to decrease oxidative stress and the IL-6 response induced by particulate matter (29). Additionally, it has effects on muscle function and lung function (16, 30). A randomized, double-blind, placebo-controlled trial conducted on COPD patients examined the effects of vitamin D supplementation. The study revealed that supplementing with vitamin D can help prevent moderate or severe exacerbations (31). This suggests that vitamin D deficiency in COPD patients might increase the risk of such exacerbations. In another clinical study focusing on vitamin D treatment for acute exacerbations of COPD (AECOPD), it was observed that the vitamin D group experienced a statistically significant improvement in Health Related Quality of Life compared to the placebo group. However, rehospitalization and mortality rates did not show significant differences between the two groups (32). In our study, we observed that vitamin D supplementation could effectively reduce in-hospital and ICU mortality in COPD patients admitted to the ICU. The discrepancy between our study findings and the previous one could be attributed to two main factors. Firstly, the previous study had a small sample size of only 62 patients, with 30 in the vitamin D group and 32 in the placebo group. Secondly, our study specifically focused on COPD patients admitted to the ICU, who tend to be more critically ill. Interestingly, we discovered a significant reduction in mortality among female COPD patients following vitamin D treatment. Furthermore, an observational study revealed that female COPD patients had higher levels of vitamin D compared to male patients, which might be associated with long-term smoking (33). Based on these findings, we conducted an analysis suggesting that female COPD patients in the ICU are more likely to achieve normal 25(OH)D concentration through supplementation, thereby reducing mortality rates.

To the best of our knowledge, this is the first study to examine the relationship between vitamin D supplementation and the prognosis of COPD patients admitted to the ICU. Various statistical methods were used to ensure the stability of the results. The large number of samples in the MIMIC-IV database also provided a solid foundation for our study. Of course, this study also had certain limitations. Firstly, as a retrospective cohort study, we cannot obtain the level of vitamin D in administration and the trends after treatment, which could reveal more information. Secondly, although we tried our best to balance confounders, some potential confounding bias remains. Thirdly, our study focused only on whether COPD patients were supplemented with vitamin D. Therefore, specific and optimal vitamin D doses need to be explored in future prospective studies.

## Conclusion

Vitamin D supplementation may reduce in-hospital and ICU mortality in patients with COPD in the ICU. Vitamin D may be more beneficial for female with COPD. Vitamin D is an inexpensive and safe drug, and so further clinical trials should be conducted to provide more-solid evidence on whether it improves the prognosis of ICU hospitalized COPD patients.

## Data availability statement

Publicly available datasets were analyzed in this study. This data can be found at: https://physionet.org/content/mimiciv.

## Ethics statement

The studies involving humans were approved by Institutional Review Board of the Massachusetts Institute of Technology and Beth Israel Deaconess Medical Center. The studies were conducted in accordance with the local legislation and institutional requirements. The ethics committee/institutional review board waived the requirement of written informed consent for participation from the participants or the participants’ legal guardians/next of kin because this is a publicly available anonymised database.

## Author contributions

QH: Conceptualization, Data curation, Methodology, Writing – original draft, Writing – review & editing. SH: Formal analysis, Investigation, Project administration, Supervision, Writing – review & editing. JX: Resources, Software, Validation, Visualization, Writing – review & editing. YG: Formal analysis, Methodology, Project administration, Supervision, Writing – review & editing. CL: Conceptualization, Investigation, Methodology, Project administration, Resources, Visualization, Writing – review & editing.
